# A variant of *RAG1* gene identified in severe combined immunodeficiency: a case report

**DOI:** 10.1186/s12887-022-03822-0

**Published:** 2023-02-03

**Authors:** Xinping Zhang, Xiayan Kang, Meiyu Yang, Zili Cai, Yulei Song, Xiong Zhou, Jianshe Cao, Chengjuan Wang, Kang Huang, Yani Peng, Jie He, Zhenghui Xiao

**Affiliations:** Department of Pediatric Intensive Care Unit of Hunan Children’s Hospital, Changsha, Hunan People’s Republic of China

**Keywords:** RAG deficiency, severe combined immunodeficiency, whole-exome sequencing, missense variant

## Abstract

**Background:**

The recombination-activating gene 1 (RAG1) protein is essential for the V (variable)-D (diversity)-J (joining) recombination process. Mutations in *RAG1* have been reported to be associated with several types of immune disorders. Typical clinical features driven by RAG1 variants include persistent infections, severe lymphopenia, and decreased immunoglobulin levels .

**Case presentation:**

In this study, a 2-month-24-days-old infant with recurrent fever was admitted to our hospital with multiple infections and absence of T and B lymphocytes. The infant was diagnosed with severe combined immunodeficiency (SCID). A homozygous variation c.2147G>A (NM_000448.2: exonme2: c.2147G>A (p.Arg716Gln)) was identified in the *RAG1* gene using whole-exome sequencing and Sanger sequencing. The predicted 3D structure of variant RAG1 indicated altered protein stability. Additionally, decreased expression of variant *RAG1* gene was detected at both the mRNA and protein levels.

**Conclusions:**

Our study identified a novel homozygous variant in *RAG1* gene that causes SCID. This finding expands the variant spectrum of *RAG1* in SCID and provides further evidence for the clinical diagnosis of SCID.

**Supplementary Information:**

The online version contains supplementary material available at 10.1186/s12887-022-03822-0.

## Background

Severe combined immunodeficiency (SCID) is an immune deficiency disorder presenting with heterogeneous genetic and clinical features. Patients diagnosed with SCID suffer recurrent and persistent infections during infancy, severe lymphopenia, and low or absent immunoglobulin (Ig) levels. The overall prevalence of SCID is estimated to be 1/100000-2/100000 [1, 2]. Approximately 23 % – 30 % of patients with SCID present with detectable levels of natural killer (NK) cells, but not B and T lymphocytes (B-, T-, NK+ SCID) [3].

The SCID criteria reference the Shearer et al 2014 criteria [4]. Recombination-activating gene (RAG) proteins include RAG1 and RAG2, which are crucial for Ig and T lymphocyte receptor (TCR) fragment rearrangement and are responsible for DNA recognition and cleavage at specific sequences [5]. Mutations in *RAG1* result in the complete or partial loss of recombinant enzyme activity, in unbalanced variable-diversity-joining (V(D)J) recombination, and in the impairment of T and B lymphocyte development at the early stages [6]. All these changes in biological processes lead to primary immune deficiency and inheritance patterns are usually autosomal recessive. Approximately 50 % of patients with SCID lack B/T lymphocytes (T-, B- SCID) as a result of *RAG1* and *RAG2* mutations [2]. Currently, 345 variants of *RAG1* are listed in the ClinVar Database (https://www.ncbi.nlm.nih.gov/clinvar/?term=RAG1%5Bgene%5D); of these, only 92 are reported as Pathogenic or Likely Pathogenic and the majority is missense. Only 24 variations were experimentally validated in SCID patients (Supplemental Table 1) and many *RAG1* variants still require clinical and experimental verification of their pathogenicity. Different *RAG1* variants cause variable clinical penetrance and immunophenotypes [7].

In this study, we report the case of a 2-month-old infant with SCID. Whole-exome sequencing (WES) identified a variation in *RAG1* that may lead to immunodeficiency. The 3D protein structure was affected by the mutation, and the variant gene expression was investigated.

## Case presentation

The proband was a 2-month-24-days-old infant who was admitted to the hospital with repeated fever for 8 days with no evident cause. The first pregnancy from this family resulted in a labor induction due to hydrocephalus at 6 months gestation and the second in a spontaneous abortion. The proband is the third child in this family, weighing 3100 grams at full-term delivery. He passed the physical examination at birth and had no history of illness. His parents are healthy and deny a family history of genetic diseases. At 2 months and 16 days of age, the infant developed repeated fever and was diagnosed with pneumonia and agranulocytosis. Because of persistent fever for 8 days and cough for 1 day after infection treatment, he was admitted to the hospital for further diagnosis and treatment. and recurrent fever for a long time.

**Fig. 1:**
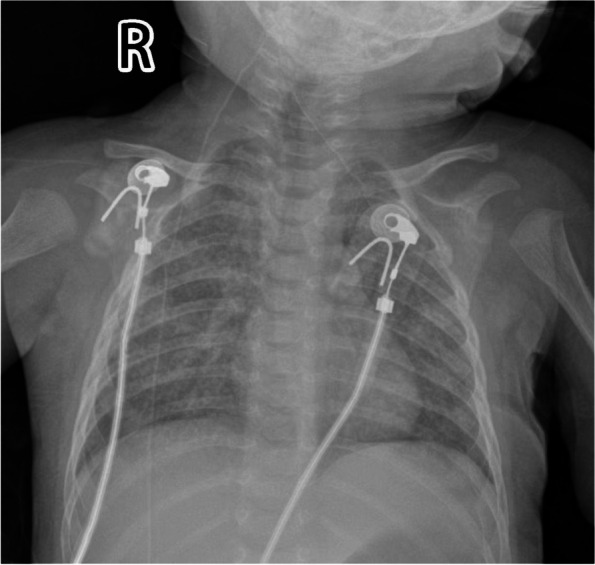
The texture of the two lungs is thickened and blurred, the inner band can be seen patchy blurred shadow, the trachea is centered, the mediastinum is centered, there is no widening, and the size of the heart's mentality is within the normal range.And the absence of a thymic shadow

Laboratory test results showed that white blood cells (WBC), polymorphonuclear (PMN), hemoglobin (HGB), red blood cells (RBC) and platelets (PLT) had decreased significantly during the hospitalization (the detailed results are shown in Table 1). Chest X-ray examinations showed centered trachea and mediastinum, no widening, and a heart size within the normal range,and the absence of a thymic shadow (the detailed results are shown in Figs. 1 and 4). Anti-infective, antibacterial, and granulocyte treatment were administered. Bone marrow cytology was normal. Detection of Ig and lymphocyte immune subsets revealed low IgG (1.72 g/L) and IgM (0.04 g/L) levels, absence of multiple lymphocytes, and a proportion of NK cells of 99.34 % (the detailed results are shown in Table 2).

**Table 1:** The results of blood during hospitalization

Date	WBC (5-12) x 10^9/L	PMN x10^9/L	LY x10^9/L	RBC (3.5-5.5)10^12/L	HGB (110-160) g/L	PLT (100-400) x 10^9/L
2020.1.09	0.61	0.30	0.1	3.44	95	256
2020.1.10	1.14	0.71	0.17	3.11	88	232
2020.1.12	2.78	2.06	0.13	2.57	72	188
2020.1.14	0.94	0.46	0.3	2.48	68	110
2020.1.16	1.29	0.73	0.18	3.3	93	19

**Table 2:** Peripheral blood analysis of proband at time of initial presentation (at age of 2-month-old) is consistent with SCID

	Patient	normal
Lymphocyte populations % (cells/μL)		
Total T cells (CD3+ CD19-)	0.00	64-73
Total B cells (CD3- CD19+)	0.00	14-21
Helper / Inducible T cells (CD3+ CD4+)	0.00	29-36
Suppressor/cytotoxic T cells (CD3+ CD8+)	0.00	24-34
Nature killer cells (CD3- CD16+ CD56+)	99.34	11-23
Immunoglobulins		
IgA (g/L)	0.07	0.05-0.41g/L
IgE (IU/mL)	<5.00	<15IU/ML
IgG (g/L)	1.72	3.2-7.2g/L
IgM (g/L)	0.04	0.23-0.91g/L

The patient also presented with diarrhea, low electrolyte sodium, and cytomegalovirus infection. Brain magnetic resonance imaging showed that the frontotemporal extracerebral space on both sides was slightly widened, and bilateral sulci fissure of the cerebral hemisphere were widened and deepened. He suffered liver damage in the later period of the treatment, and his transaminase levels increased to 425.90 IU/L (alanine aminotransferase) and 1172.00 IU/L (aspartate aminotransferase). The infant was diagnosed with primary immunodeficiency. The patient presented with severe immune system deficiency, which did not improve upon treatment, and was discharged from the hospital. He did not receive further treatment and died shortly after hospital discharge.

### Missense variation in *RAG1* gene

**Fig. 2:**
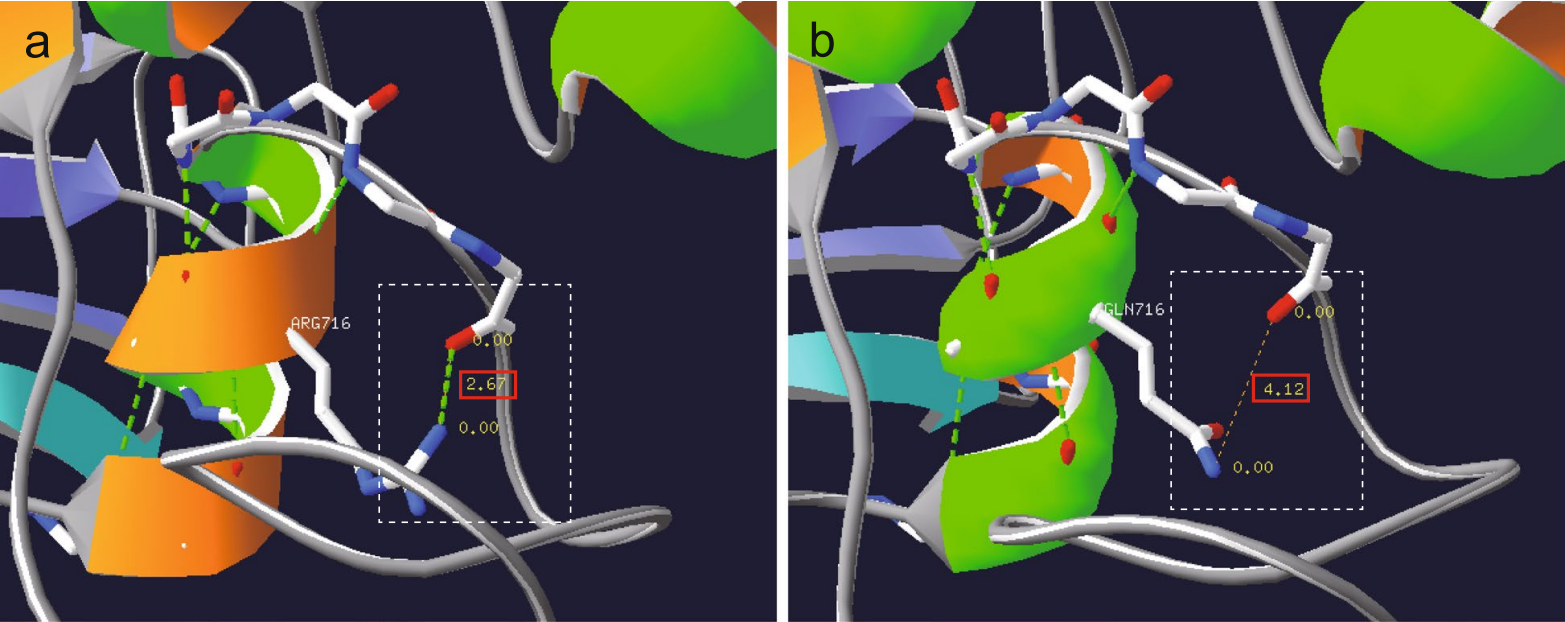
Schematic protein structure of RAG1 and species conservation analysis. **a** Pedigree of the family with RAG1 deficiency. Diagonal up arrow present proband. **b** Sanger sequencing validation of RAG1 mutation in proband and his parents. The red square confirms RAG1 mutation site. **c** Multiple species and mutation sequences alignments of RAG1 protein. The mutated site was colored and it was conserved in several species. **d** Schematic representation of the RAG1 protein domains. The full length of RAG1 protein is 1043 amino acids. It contains the RING finger domain, zinc-finger domain (ZF), HBR/NBR domain, and V(D)J recombination-activating region. The mutation-produced amino acid 716 changed from arginine to glutamine annotate as p.R716Q

WES was performed to identify potential pathogenic variants, which contributes to diagnosis confirmation. A homozygous variant c.2147G>A [NM_000448.2: exonme2: c.2147G>A (p.Arg716Gln)] in the *RAG1* gene was detected. The evidence for pathogenicity based on the ACMG guidelines was “PM2_supporting” and “PP3” and the variation category was defined as “uncertain significance.” PP4 evidence was also arguable met. The variant was inherited from the parents who were heterozygous carriers (Fig. 2a). We further confirmed the variant using Sanger sequencing (Fig. 2b). This variant was listed in the ClinVar database (https://www.ncbi.nlm.nih.gov/clinvar/mutation/418448/) as “likely pathogenic” and was described in a multi-institutional study in India [8]; however, there were no reports on its experimental verification. The allele frequency of c.2147G>A is 6.57e^-6^ according to the public database gnomAD (http://gnomad.broadinstitute.org), suggesting that the homozygous variant of p.Arg716Gln is extremely rare. The variant was predicted to be disease-causing according to multiple software (Table 3). We analyzed the domain arrangement of the RAG1 protein and found that the mutation is located in the V(D) J recombination-activating region, which is important for protein function (Fig. 2d). Species conservation analysis showed that this region is highly conserved in multiple species (Fig. 2c).

**Table 3:** Prediction of the pathogenicity of the p.Arg716Gln mutation in the RAG1

Software	Prediction	Score
SIFT	Damaging	0
Provean	Deleterious	-2.5
Polyphen	Probably damaging	0.99
Mutation Assessor	High functional impact	3.705
Mutation Taster	Disease causing	1
FATHMM	Damaging	-2.85

### Protein modeling and stability analysis

**Fig. 3:**
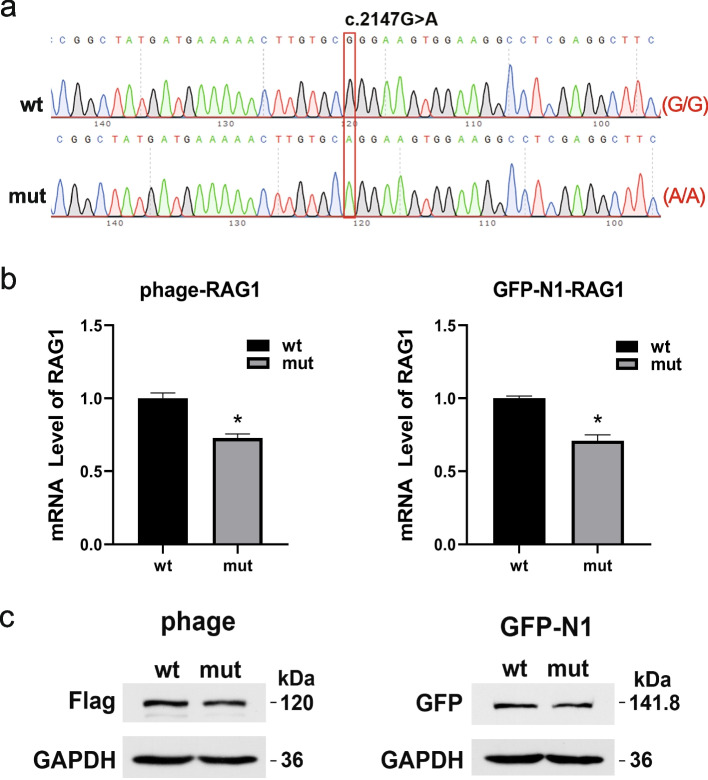
RAG1 protein 3D structure of the WT and mutation in p.Arg716Gln. **a** WT protein. **b**. Mutated protein. The white dotted box showed the hydrogen bonding (the green dotted line) disappeared and the distance between 716 and 722 amino acids was larger from 2.67Å to 4.12Å

The Swiss-Pdb Viewer was used to predict and compare the structures of wild-type (WT) and mutant RAG1 [9]. The 3D protein structure of RAG1 used as a template was 89.9 % similar to that of the target protein. The arginine-to-glutamine substitution at position 716 resulted in the disruption of a hydrogen bond with lysine at position 722 (Fig. 3). The distance between residues 716 and 722 increased from 2.67 Å to 4.12 Å in the variant protein, which may impact its stability and function.

### The mutation affects *RAG1* expression

**Fig. 4:**
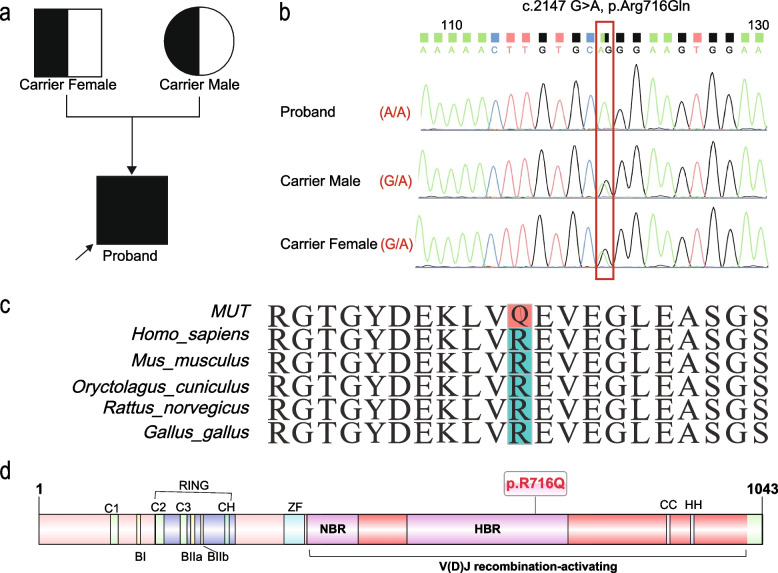
The expression of RAG1 in c.2147G>A (p.Arg716Gln) mutation. **a** WT and mutant fragments were cloned into pEGFP and phage expression vectors. Genomic DNA samples were collected after 293T cells transfection 48 hours, and mutation was confirmed by Sanger sequencing. The result showed recombinant vectors successfully introduced WT (upper) and mutant fragments (bottom). The red box showed the base mutation. **b** The relative mRNA levels of *RAG1* were performed by q-PCR analysis. Obviously low expression of *RAG1* in both pEGFP-RAG1-mut and phage-RAG1-mut samples compared to WT. Data are mean ± SD, * *P*<0.05. **c** The protein levels of RAG1 in WT and mutation. Western blot examination showed RAG1 slightly reduced in mutated groups (at the upper right band). However, the expression of internal protein GAPDH did not changed

To further investigate the impact of the mutation on protein function, recombinant expression plasmids (pEGFP-N1-RAG1-wt/mut and phage-RAG1-wt/mut) were constructed and transfected into 293T cells. Samples were collected 48 h after transfection. The c.2147G>A (p.Arg716Gln) variation was successfully introduced into recombinant expression plasmids and validated by sequencing (Fig. 4a). Quantitative polymerase chain reaction (qPCR) showed that the expression of the variant gene was significantly lower than that of the WT, using the two sets of plasmids. The levels of variant mRNA decreased to 72.63 % and 70.9 % compared with those of WT mRNA using the phage and pEGFP-N1 plasmids, respectively (Fig. 4b). Western blotting showed that the protein levels of mutant RAG1 were lower compared with those of WT RAG1 using both phage (120 kd) and pEGFP-N1 (141.8 kd) plasmids (Fig. 4c). Overall, these results indicate a lower expression of variant *RAG1* at both mRNA and protein levels.

## Materials and methods

### Whole-exonme sequencing (WES)

The patient’s parents gave informed consent and agreed to participate in this study. To further explore the etiology of the disease, blood samples were drawn from the proband and his parents after obtaining consent. Sample sequencing, database filtering, and variant interpretation proceeded subsequently. Variant pathogenicity was predicted using SIFT [10], Polyphen-2 [11], Provean [12], Mutation Assessor [13], Mutation Taster [14], and FATHMM [15] software. The pathogenic classification was determined according to the ACMG guidelines.

### 3D protein structure modeling

Molecular modeling analysis was performed to estimate the impact of p.Arg716Gln on the protein structure. The structures of WT and variant RAG1 proteins were predicted using the Swiss-Model program. The Swiss-Pdb Viewer software was used for analyzing the structure of WT and mutant RAG1 proteins.

### Cell culture and transfection

The 293T cells were cultured in DMEM medium with 10 % fetal bovine serum. We cloned the WT and variant fragments into two eukaryotic expression vectors (pEGFP-N1-RAG1-wt/mut and phage-RAG1-wt/mut). The primer sequences of phage-RAG1 (PHAGE-RAG1-SalI-F, PHAGE-RAG1-SalI-R) are shown in Supplemental Table 2. The plasmids were transfected into 293T cells using the Lipo2000 Transfection Reagent (Lipofectamine^TM^ 2000,ThermoFisher Scientific, Waltham,MA,USA) according to the manufacturer’s protocol.

### Quantitative real-time PCR

The 293T cells were collected 48 hours after transfection. Total RNA was extracted using Trizol. cDNA was synthesized after digestion of DNA using the Prime Script RT Reagent kit with gDNA Eraser (RR047A, TaKaRa). The difference in mRNA expression between WT and mutant genes was detected by qPCR analysis, using the SYBR Green Realtime PCR Master Mix (QPK-201, Toyobo) in a CFX Connect Real-Time PCR Systems (BioRad). The two-pair primers used were RAG1-QPCR-F and RAG1-QPCR-R and the detail of primer sequences was shown in Supplemental Table 2. The statistical method used for analysis of qPCR data was the t-test.

### Western blot

We collected cells to detect the protein expression levels of the WT and mutant samples. RIPA Lysis Buffer (C1053, PPLYGEN) was used to extract the total proteins. A BSA kit (20201ES76, YEASEN) was used to determine the protein concentration and then perform protein denaturation treatment. The total protein was loaded per lane on an SDS-PAGE gel and transferred to a polyvinylidene difluoride membrane. The membrane was incubated with primary antibodies overnight at 4 °C after blocking with 5 % skim milk for 1 h. The details of the primary antibodies used in this study are described in Supplemental Table 3.

## Discussion and conclusions

SCID is a group of genetic diseases characterized by cell and humoral immune function defects, which cause severe dysfunction of the immune system [16]. Patients often have early clinical manifestations, as serious infections and dysplasia, including pneumonia, otitis media, skin infections, or disseminated BCG infection. Some patients may lack typical symptoms. Patients with SCID who are not diagnosed in time often died during the first two years of life [17]. A typical clinical feature of SCID is the absence of T lymphocytes in the blood and lymphoid tissues [18]. SCID is divided into two main classes: with or without B lymphocytes (B+/- SCID).

Human RAG includes RAG1 and RAG2, which were necessary for the rearrangement of Ig and TCR gene fragments [19]. Mutations in *RAG1* make the encoded recombinase activity completely or partially lost and the development of T and B lymphocytes blocked in the early stage, finally leading to primary immunodeficiency. RAG variations in humans have been associated with a broad range of phenotypes such as SCID, combined cellular and humoral immune defects with granulomas [20], and Omenn syndrome. However, the severity of the clinical phenotype of RAG deficiency correlates with the level of recombination activity of the mutant protein [6].

Typical SCID is caused by mutations in the *RAG1* gene and has a specific immunologic phenotype, with negative T and B lymphocytes and positive NK cells, which was first investigated in 1996 [3]. In this study, we report a SCID patient with a homozygous variant c.2147 G>A variant in the *RAG1* gene detected by WES. The patient presented with typical clinical features of T-, B-, and NK+ SCID. He presented with gastrointestinal and respiratory infections similar to other T-, B-, NK+ SCID patients [21–23]. Different from the Omenn syndrome, he had marked lymphopenia, low serum Ig levels, and very low or even absent T and B cells; these are typical clinical manifestations of T-, B-, and NK+ SCID [24].

The human RAG1 protein contains 1043 amino acids and is composed of non-core and core regions. We also observed the nonamer-binding region (NBR), the heptamer-binding region (HBR), and the zinc finger in the protein structure (Fig. 2d). A systematic study based on 79 *RAG1* mutations has shown that the recombination activity of the mutated proteins is significantly related to the severity of the phenotype, and that mutation recombination activity in NBR/HBR is significantly lower than that in other regions [6]. The p.Arg716Gln variation identified in this study was located in the core region and HBR (amino acids 531–763), possibly affecting the activity of the protein. Subsequently, we analyzed the 3D structure of WT and mutant proteins. The results showed that the mutation resulted in the loss of a hydrogen bond between residues 716 (the substituted amino acid in our variant) and 722. The p.Arg716Gln variation is clustered around the active site and may alter the structure of the catalytic center or change its DNA-binding properties [25].

Although there are extensive clinical studies of patients with SCID carrying *RAG1* mutations and their clinical phenotype has been clarified, experimental studies on the variation are still limited. To the best of our knowledge, only 24 *RAG1* variations have been experimentally verified. The variations generally affect the expression of RAG1 [6], especially the RAG recombination activity [26]. In our study, cell transfection experiments were performed to further explore the impact of the variation on gene expression. The results showed slightly decreased mRNA and protein levels in the mutated group (Fig. 3). RAG1 missense variants may affect the protein function by changing the protein structure rather than its expression. A study by Notarangelo [26] experimentally verified 51 RAG1 missense variants, of which 33 were tested for protein expression. The results showed that approximately 30 % of the variants (11/33) retained >75 % of their protein expression levels. These mutations may affect the function of the protein by reducing its recombination activity or changing the stability of important functional domains. The mutation R716Q is located near the conserved catalytic amino acids D603, D711, and E965 and, theferore, may modify the structure of the catalytic center and the DNA-binding ability.

In conclusion, we reported a typical SCID patient with a homozygous *RAG1* variant, which was inherited from his parents. Our report expands the spectrum of known *RAG1* variants in Chinese patients with SCID. These results also demonstrated the utility of WES for identifying variants and differential etiology.

## Supplementary Information

**Additional file 1:** **Supplemental Table 1.** References for functional validation of RAG1 variation in SCID patients, **Supplemental Table 2.** Primer sequence of plasmid construct and q-PCR, **Supplemental Table 3.** Antibody information for Western Blot.

## Data Availability

All the data generated and/or analyzed during this study available from the corresponding author on reasonable request. The datasets generated and/or analysed during the current study are available in the Clinvar DATE repository, (Submission ID:SUB12334160)

## Abbreviations

HBR: heptamer-binding region
Ig: immunoglobulin
NBR: nonamer-binding region
NK: natural killer
qPCR: quantitative polymerase chain reaction
RAG: recombination-activating gene
SCID: severe combined immunodeficiency
TCR: T lymphocyte receptor
V(D)J: variable-diversity-joining
WES: whole-exome sequencing
WT: wild-type.
