# Supplementary material for: A variant of RAG1 gene identified in severe combined immunodeficiency: a case report
Source: BMC Pediatr. 2023 Feb 3;23:56. doi: 10.1186/s12887-022-03822-0 (PMC9896705; doi:10.1186/s12887-022-03822-0)
Supplement: Supplementary file 1 — Additional file 1: Supplemental Table 1. References for functional validation of RAG1 variation in SCID patients, Supplemental Table 2. Primer sequence of plasmid construct and q-PCR, Supplemental Table 3. Antibody information for Western Blot. [file 12887_2022_3822_MOESM1_ESM.zip › Supplemental MaterialsR8.docx]

Supplemental Materials

Supplemental Table 1. References for functional validation of RAG1 variation in SCID patients.

| Case No. | Varitaion | Type | Reference |
| --- | --- | --- | --- |
| 1 | p.H103Y | leaky SCID | (Khan et al. 2017) |
| 2 | p.R404Q | SCID | (Hedayat et al. 2014) |
| 3 | p.C358Y | leaky SCID | (Abolhassani et al. 2014) |
| 4 | p.A444V | SCID | (Lee et al. 2014) |
| 5 | p.M1006V | SCID | (Lee et al. 2014) |
| 6 | p.R764P | SCID | (Lee et al. 2014) |
|  | p.R449K |  |  |
|  | p.R404Q |  |  |
| 7 | p.R394Q | SCID | (Lee et al. 2014) |
| 8 | p.T403P | SCID | (Lee et al. 2014) |
| 9 | p.V475AfsX17 | SCID | (Lee et al. 2014) |
| 10 | p.L411P | SCID | (Lee et al. 2014) |
| 11 | p.S626X | SCID | (Lee et al. 2014) |
| 12 | c.631delT | SCID | (Noordzij et al. 2000) |
| 13 | p.E770K | Atypical SCID | (Asai et al. 2011) |
| 14 | p.R561H | SCID | (Ehl et al. 2005) |
| 15 | c.2113delC | SCID/OS | (Wada et al. 2005) |
| 16 | c.256-257delAA | atypical SCID | (Sharapova et al. 2013) |
|  | p.A444V |  |  |
| 17 | p.R897X | atypical SCID | (Kumaki et al. 2001) |
|  | p.R559S |  |  |
| 18 | p.Z722K | SCID | (Schwarz et al. 1996) |
| 19 | p.Y938* | SCID | (Schwarz et al. 1996) |
| 20 | p.A156V | SCID | (Schwarz et al. 1996) |
| 21 | p.R624H | SCID | (Schwarz et al. 1996) |
| 22 | p.R394Q | SCID | (Notarangelo et al. 2016) |
| 23 | p.D429G | SCID | (Notarangelo et al. 2016) |
| 24 | p.A444V | SCID | (Notarangelo et al. 2016) |

Supplemental Table 2. Primer sequence of plasmid construct and q-PCR

|  | Primer name | Primer sequence |  |
| --- | --- | --- | --- |
| plasmid construct | PHAGE-RAG1-SalI-F | TGACGTCGACCATGGCAGCCTCTTTCCCACC | |
|  | PHAGE-RAG1-NotI-R | CGACGCGGCCGCGAAATTCCATTGAATCTTGGC | |
| q-PCR | RAG1-QPCR-F | GGGAGACGTGAGTGAGAAGC |  |
|  | RAG1-QPCR-R | TCGTGGTCAGACTCATCTGC |  |

Supplemental Table 3. Antibody information for Western Blot

| Product code | Primary antibody | species | kDa | dilution ratio | company |
| --- | --- | --- | --- | --- | --- |
| 2056 | Flag | M. |  | 1:1500 | QB |
| 2118S | GAPDH | R. | 36 | 1:1000 | CST |
| CSB-MA000051 | GFP | M |  | 1:1000 | CUSABIO |

Supplementary Reference

Abolhassani, H., N. Wang, A. Aghamohammadi, N. Rezaei, Y. N. Lee, F. Frugoni, L. D. Notarangelo, Q. Pan-Hammarström, and L. Hammarström. 2014. 'A hypomorphic recombination-activating gene 1 (RAG1) mutation resulting in a phenotype resembling common variable immunodeficiency', *The Journal of allergy and clinical immunology*, 134: 1375-80.

Asai, E., T. Wada, Y. Sakakibara, A. Toga, T. Toma, T. Shimizu, S. Nampoothiri, K. Imai, S. Nonoyama, T. Morio, H. Muramatsu, Y. Kamachi, O. Ohara, and A. Yachie. 2011. 'Analysis of mutations and recombination activity in RAG-deficient patients', *Clin Immunol*, 138: 172-7.

Ehl, S., K. Schwarz, A. Enders, U. Duffner, U. Pannicke, J. Kühr, F. Mascart, A. Schmitt-Graeff, C. Niemeyer, and P. Fisch. 2005. 'A variant of SCID with specific immune responses and predominance of gamma delta T cells', *J Clin Invest*, 115: 3140-8.

Hedayat, M., M. J. Massaad, Y. N. Lee, M. E. Conley, J. S. Orange, T. K. Ohsumi, W. Al-Herz, L. D. Notarangelo, R. S. Geha, and J. Chou. 2014. 'Lessons in gene hunting: a RAG1 mutation presenting with agammaglobulinemia and absence of B cells', *The Journal of allergy and clinical immunology*, 134: 983-5.e1.

Khan, T. A., A. Iqbal, H. Rahman, O. Cabral-Marques, M. Ishfaq, and N. Muhammad. 2017. 'Novel RAG1 mutation and the occurrence of mycobacterial and Chromobacterium violaceum infections in a case of leaky SCID', *Microb Pathog*, 109: 114-19.

Kumaki, S., A. Villa, H. Asada, S. Kawai, Y. Ohashi, M. Takahashi, I. Hakozaki, E. Nitanai, M. Minegishi, and S. Tsuchiya. 2001. 'Identification of anti-herpes simplex virus antibody-producing B cells in a patient with an atypical RAG1 immunodeficiency', *Blood*, 98: 1464-8.

Lee, Yu Nee, Francesco Frugoni, Kerry Dobbs, Jolan E. Walter, Silvia Giliani, Andrew R. Gennery, Waleed Al-Herz, Elie Haddad, Francoise LeDeist, Jack H. Bleesing, Lauren A. Henderson, Sung-Yun Pai, Robert P. Nelson, Dalia H. El-Ghoneimy, Reem A. El-Feky, Shereen M. Reda, Elham Hossny, Pere Soler-Palacin, Ramsay L. Fuleihan, Niraj C. Patel, Michel J. Massaad, Raif S. Geha, Jennifer M. Puck, Paolo Palma, Caterina Cancrini, Karin Chen, Mauno Vihinen, Frederick W. Alt, and Luigi D. Notarangelo. 2014. 'A systematic analysis of recombination activity and genotype-phenotype correlation in human recombination-activating gene 1 deficiency', *The Journal of allergy and clinical immunology*, 133: 1099-108.

Noordzij, J. G., N. S. Verkaik, N. G. Hartwig, R. de Groot, D. C. van Gent, and J. J. van Dongen. 2000. 'N-terminal truncated human RAG1 proteins can direct T-cell receptor but not immunoglobulin gene rearrangements', *Blood*, 96: 203-9.

Notarangelo, L. D., M. S. Kim, J. E. Walter, and Y. N. Lee. 2016. 'Human RAG mutations: biochemistry and clinical implications', *Nat Rev Immunol*, 16: 234-46.

Schwarz, K., G. H. Gauss, L. Ludwig, U. Pannicke, Z. Li, D. Lindner, W. Friedrich, R. A. Seger, T. E. Hansen-Hagge, S. Desiderio, M. R. Lieber, and C. R. Bartram. 1996. 'RAG mutations in human B cell-negative SCID', *Science (New York, N.Y.)*, 274: 97-99.

Sharapova, S. O., A. Migas, I. Guryanova, S. Aleshkevich, S. Kletski, A. Durandy, and M. Belevtsev. 2013. 'Late-onset combined immune deficiency associated to skin granuloma due to heterozygous compound mutations in RAG1 gene in a 14 years old male', *Hum Immunol*, 74: 18-22.

Wada, T., T. Toma, H. Okamoto, Y. Kasahara, S. Koizumi, K. Agematsu, H. Kimura, A. Shimada, Y. Hayashi, M. Kato, and A. Yachie. 2005. 'Oligoclonal expansion of T lymphocytes with multiple second-site mutations leads to Omenn syndrome in a patient with RAG1-deficient severe combined immunodeficiency', *Blood*, 106: 2099-101.
