# Supplementary figures and images for: A variant of RAG1 gene identified in severe combined immunodeficiency: a case report
Source: BMC Pediatr. 2023 Feb 3;23:56. doi: 10.1186/s12887-022-03822-0 (PMC9896705; doi:10.1186/s12887-022-03822-0)

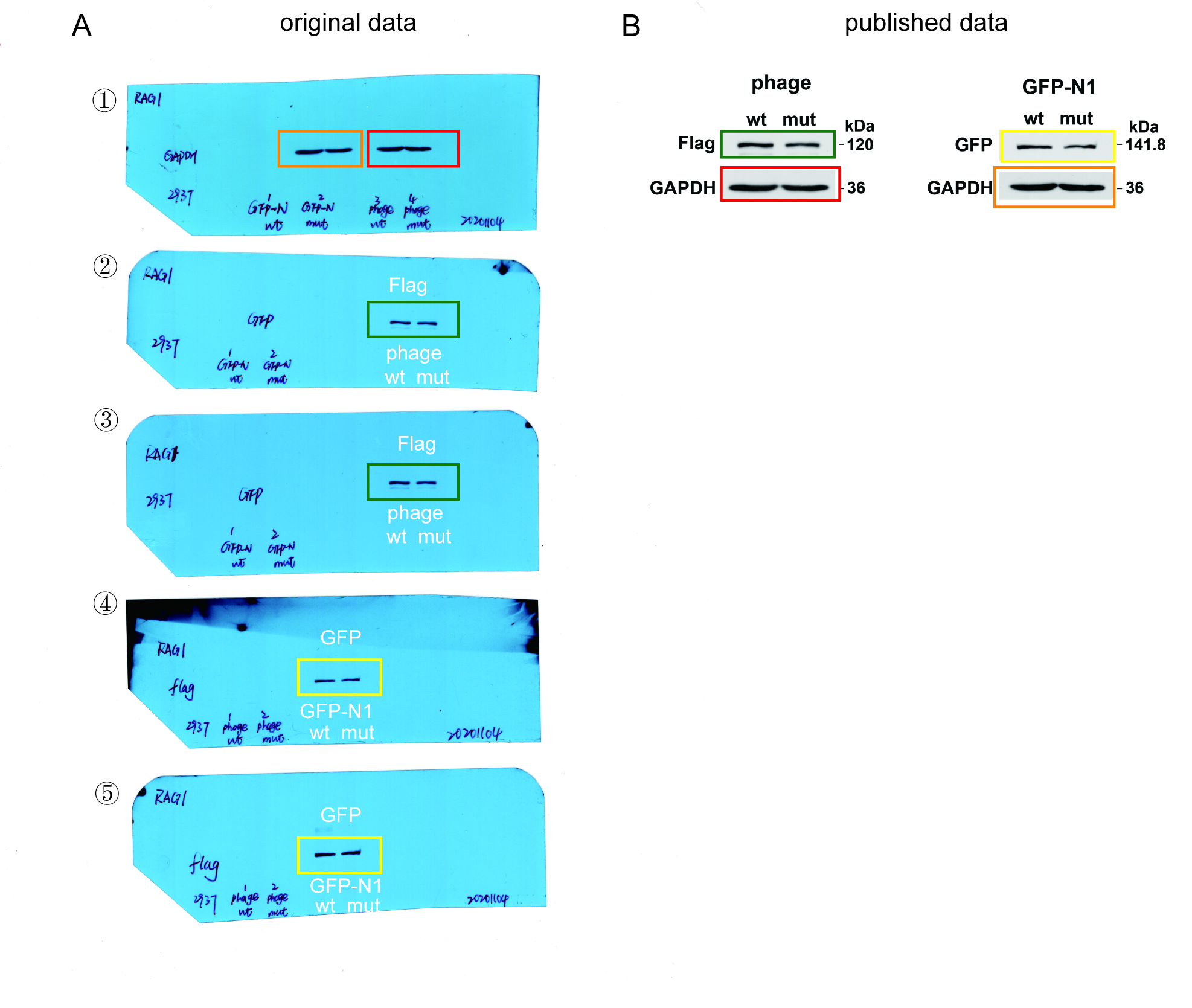

Supplement: Supplementary file 1 — Additional file 1: Supplemental Table 1. References for functional validation of RAG1 variation in SCID patients, Supplemental Table 2. Primer sequence of plasmid construct and q-PCR, Supplemental Table 3. Antibody information for Western Blot. [file 12887_2022_3822_MOESM1_ESM.zip › Supplimentary Figure 1R8.tif]
